# Screening for Latent Tuberculosis Infection in People Living with HIV: TUBHIVIT Project, a Multicenter Italian Study

**DOI:** 10.3390/v16050777

**Published:** 2024-05-14

**Authors:** Luca Pipitò, Elena Delfina Ricci, Paolo Maggi, Giuseppe Vittorio De Socio, Giovanni Francesco Pellicano, Marcello Trizzino, Raffaella Rubino, Alessandra Lanzi, Lorenzo Crupi, Ilaria Capriglione, Nicola Squillace, Giuseppe Nunnari, Antonio Di Biagio, Paolo Bonfanti, Antonio Cascio

**Affiliations:** 1 Department of Health Promotion, Mother and Child Care, Internal Medicine and Medical Specialties, University of Palermo, 90127 Palermo, Italy; luca.pipito@community.unipa.it (L.P.); marcello.trizzino@policlinico.pa.it (M.T.); raffaella.rubino@policlinico.pa.it (R.R.); 2 Infectious and Tropical Disease Unit, AOU Policlinico “P. Giaccone”, Via del Vespro 129, 90127 Palermo, Italy; 3 Fondazione ASIA Onlus, 20090 Buccinasco, Italy; ed.ricci@libero.it; 4 Infectious Diseases Unit, AORN Sant’Anna e San Sebastiano, 81100 Caserta, Italy; paolo.maggi@unicampania.it (P.M.); ilariacapriglione9@gmail.com (I.C.); 5 Unit of Infectious Diseases, Santa Maria Hospital, 06156 Perugia, Italy; giuseppe.desocio@ospedale.perugia.it (G.V.D.S.); alessandralanzi@libero.it (A.L.); 6 Infectious Diseases, G. Martino Hospital-University of Messina, 98147 Messina, Italy; gpellicano@unime.it (G.F.P.); gnunnari@hotmail.com (G.N.); 7 Infectious Diseases, San Martino Hospital Genoa, University of Genoa, 16131 Genoa, Italy; crupi88@gmail.com (L.C.); antonio.dibiagio@hsanmartino.it (A.D.B.); 8 Infectious Diseases Unit, Fondazione IRCCS San Gerardo dei Tintori, 20900 Monza, Italy; nicola.squillace@irccs-sangerardo.it (N.S.); paolo.bonfanti@unimib.it (P.B.); 9 Department of Clinical and Experimental Medicine, Unit of Infectious Diseases, ARNAS Garibaldi Hospital, University of Catania, 95122 Catania, Italy; 10 Department of Medicine, University of Milano-Bicocca, 20126 Milano, Italy

**Keywords:** tuberculosis, HIV, latent tuberculosis, TB, LTBI

## Abstract

Background: The coexistence of HIV infection and latent tuberculosis infection (LTBI) presents a significant public health concern due to the increased risk of tuberculosis (TB) reactivation and progression to active disease. The multicenter observational cohort study, TUBHIVIT, conducted in Italy from 2017 to 2023, aimed to assess the prevalence of LTBI among people living with HIV (PLHIV) and their outcomes following LTBI screening and therapy initiation. Methods: We performed a prospective study in five referral centers for HIV care in Italy. PLHIV who consented Tto participate underwent QuantiFERON-TB Gold Plus and clinical, microbiological, and radiological assessments to exclude subclinical tuberculosis, as opportune. PLHIV diagnosed with LTBI who started chemoprophylaxis were followed until the end of therapy. Results: A total of 1105 PLHIV were screened for LTBI using the QuantiFERON-TB Gold Plus test, revealing a prevalence of 3.4% of positive results (38/1105). Non-Italy-born individuals exhibited a significantly higher likelihood of testing positive. Thirty-one were diagnosed with LTBI, 1 showed active subclinical TB, and 6 were lost to follow-up before discriminating between latent and active TB. Among the PLHIV diagnosed with LTBI, 83.9% (26/31) started chemoprophylaxis. Most individuals received 6–9 months of isoniazid-based therapy. Of the 26 PLHIV commencing chemoprophylaxis, 18 (69.2%) completed the therapy, while 3 discontinued it and 5 were still on treatment at the time of the analysis. Adverse events were observed in two cases, while in one case the patient refused to continue the treatment.

## 1. Introduction

Tuberculosis (TB) is one of the leading causes of morbidity and mortality among people living with HIV (PLHIV) worldwide [1]. Worldwide, an estimated 10.6 million people developed TB in 2022 and TB caused an estimated 1.30 million deaths. Among all incident cases of TB in 2022, 6.7% were PLHIV and there were 167,000 deaths from TB among PLHIV, which were officially classified as deaths from HIV/AIDS [1]. World Health Organization (WHO) guidelines recommend regular screening for active and latent TB infection (LTBI) in all PLHIV, including those on ART, using a combination of clinical assessment, symptom screening, and diagnostic tests, as appropriate [1]. Furthermore, WHO guidelines also recommend the treatment of latent infections to reduce the risk of progression to active disease, irrespective of the degree of immunosuppression. PLHIV are more vulnerable to TB infection due to their compromised immune systems and are at increased risk of TB reactivation compared to the HIV-negative population [1]. Various studies have reported that HIV infection might lead to a 10–110 times higher risk of LTBI reactivation [2]. A recent metanalysis regarding screening for TB infection in PLHIV showed that preventive treatment significantly reduced the incidence of TB in this group [3]. The pooled incidence rate in the preventive treatment group was 6 cases per 1000 person-years, while the pooled incidence rate in the non-preventive treatment group was 65 cases per 1000 person-years [3]. Several screening methods can be used in PLHIV, including symptom-based screening, c-reactive protein, tuberculin skin testing (TST), interferon-gamma release assays (IGRAs), and chest X-rays. Compared to TST, the IGRA test is not positive in vaccinated people, has a higher sensitivity in patients with lower CD4 counts, and greater specificity in those from areas with a low incidence of TB [4]. In asymptomatic PLHIV, with a positive result for TST or IGRAs, and negative medical history for previous TB treatment or a previous Bacillus Calmette–Guérin vaccination, subclinical TB should be excluded by chest X-rays, computed tomography (CT), and/or the microscopic and molecular examination of sputum, as opportune [5]. In fact, LTBI is defined as a state of persistent immune response to stimulation by *Mycobacterium tuberculosis* antigens with no evidence of clinically manifest active TB [1]. If active TB is excluded, therapy for LTBI should be initiated. In Italy, three regimens for latent TB are usually considered: 4 months of once-daily rifampin, 3 months of once-daily isoniazid plus rifampin, or isoniazid administered once daily for 6 or 9 months. Although rifampin is the drug of choice, given the known activity on dormant cells independent of the cell cycle, less toxicity, and more adherence due to short regimen duration [6], its use is severely limited by pharmacological interactions, especially in PLHIV, on antiretroviral therapy [7]. In addition, isoniazid and rifampin can cause hepatotoxicity; therefore, a chemistry follow-up is necessary. These factors limit the clinician in initiating therapy for LTBI; there is no common consensus concerning screening and treating PLHIV for LTBI, despite WHO recommendations, as has emerged in a Italian survey regarding newly diagnosed PLHIV [8]. In Italy, few data are published about the prevalence of active TB and LTBI [8,9]. Italy is considered a low-TB-incidence country, with an estimated 4000 new cases per year (<10 per 100,000 inhabitants), with 7.5 new cases per 100,000 inhabitants from 2011 to 4 new cases per 100,000 inhabitants in 2020 [9]. In Sicily, 5239 admissions for TB occurred between 2009–2021, with 2.8% of these regarded as PLHIV. In addition, HIV is an independent predictor of mortality [10]. However, no data were reported for LTBI [10]. Our study aims to describe the prevalence of LTBI in PLHIV and their management in a multicenter cohort of PLHIV. 

## 2. Materials and Methods

The TUBHIVIT project is a multicenter observational cohort study initiated in 2019. The study aims to gather data on the prevalence of LTBI among PLHIV in participating Italian infectious disease centers from 2017 to 2023. Additionally, PLHIV who have initiated treatment for LTBI were prospectively followed. These centers adhere to the recommendations outlined in international guidelines [7], typically offering screening to PLHIV, often at the time of HIV diagnosis, using intradermal testing according to the Mantoux method (considering an induration >5 mm as positive) or the QuantiFERON test. In individuals testing positive on QuantiFERON, a chest X-ray or CT scan and sputum smear microscopy are typically conducted to rule out subclinical TB. All PLHIV attending the HIV outpatient clinic in the centers from December 2017 until December 2023 were eligible for screening, except those who were currently being investigated or treated for active TB, and PLHIV who had ever received treatment for suspected or confirmed TB. Inclusion criteria encompass PLHIV aged over 18 years who consented to participate and underwent screening for LTBI using interferon-gamma release assays (IGRA). QuantiFERON-TB Gold Plus (QFT) was the IGRA test used. Retrospective data, collected from 1 December 2017 until the start of the study, were incorporated. The clinical data, collected from case report forms, included demographic information, such as sex, age, ethnicity, comorbidities (up to three per patient), whether the patients were men having sex with men, as well as information about alcohol use and drug use. Laboratory data comprised the most recent previous CD4+ T cell count and nadir CD4+ T cell count for all screened PLHIV. Additionally, ART history, aspartate aminotransferase (AST), alanine aminotransferase (ALT), and bilirubin levels were recorded at baseline and were prospectively collected biweekly for the first month, and then monthly in those who initiated therapy for LTBI. Adverse events (AEs) were prospectively collected upon clinical observation. Reasons for failure to initiate therapy and failure to complete therapy were also documented.

### Statistical Analysis

Data were described using mean and standard deviation (SD) for normally distributed continuous variables; median, and interquartile range (IQR) for not normally distributed continuous variables; and frequency (%) for categorical and ordinal variables. Distribution normality was assessed using the graphical quantile–quantile method. Baseline differences were tested using the analysis of variance for means, the non-parametric Mann–Whitney test for medians, and proportion comparisons were performed using the chi-square test.

## 3. Results

**Figure 1:**
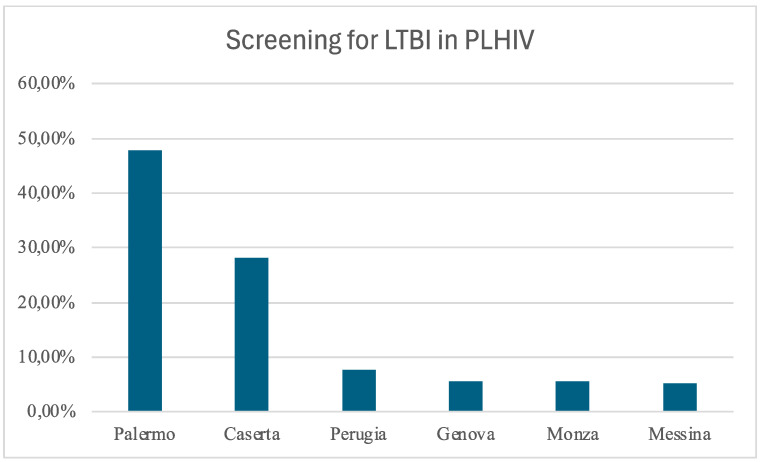
Distribution of the enrolled patients among the centers: Palermo, Policlinico Giaccone; Caserta, Sant’Anna and Sebastiano Hospital; Perugia, Santa Maria Hospital; Genova, IRCCS San Martino; Monza, IRCCS San Gerardo; Messina, G. Martino Hospital.

The study included 1105 PLHIV who underwent TB screening, all via the IGRA test. The contributions of each center are depicted in Figure 1. 

**Figure 2:**
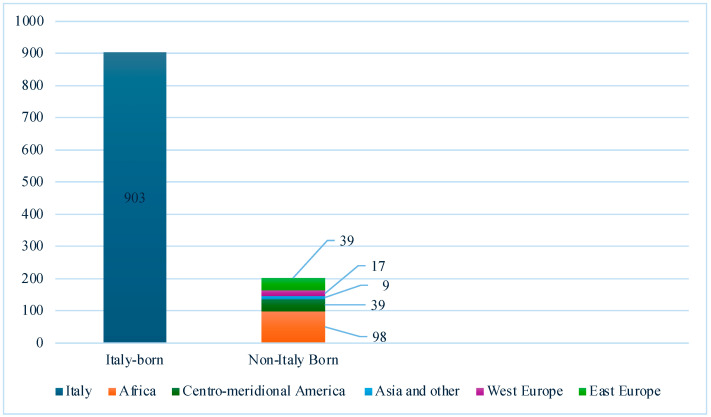
Absolute frequencies of PLHIV screened for LTBI in Italian centers (n = 1105).

Over half (75.2%) of the cohort were men and 18.3% were non-Italy-born PLHIV. Absolute frequencies of the country of origin are visually represented in Figure 2.

The median age of the participants was 49 years (IQR 40–56), with a median CD4 cell count of 618 cells/μL (IQR 426–840). Among them, 11.4% reported drug addiction, 24.8% were men who have sex with men (MSM), and 4.1% exhibited alcohol abuse. The diagnosis of HIV infection had been made a median of 7.2 years earlier (IQR 2.3–17.3); 27.7% of the screened PLHIV had at least one comorbidity, predominantly cardiovascular diseases (22.0%). The QFT TB Gold Plus test resulted in 1056 negative results, 38 positive results (prevalence 3.4%), and 11 indeterminate results (1.0%). Of the latter, only 1 had a CD4 cell count < 200. A comparison of demographic and clinical characteristics between those with positive results suggestive of latent tuberculosis infection (LTBI) and those with negative results is presented in Table 1.

**Figure 3:**
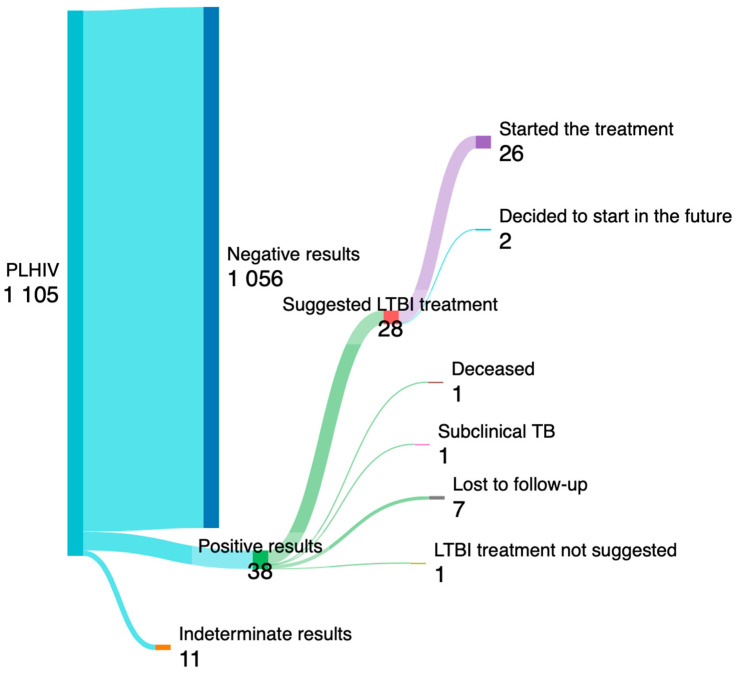
The Sankey plot illustrates the process screening of PLHIV for LTBI. Tthe number of PLHIV who started the treatment for LTBI is shown at the end.

One individual was found to have active subclinical TB disease during clinical and radiological assessments. Six PLHIV with positive results were lost to follow-up before discriminating the type of TB (latent of active infection), while 31 PLHIV out of 1087 people who completed the screening (2.8%) showed latent tuberculosis. Non-Italy-born individuals were significantly more likely to test positive for IGRA (6.15% versus 2.13%, *p* = 0.002), with the majority of those testing positive originating from Africa (7/12, 58.3%). Sex and age were similar between PLHIV with positive and negative results, while the proportion of PLHIV with negative results showed a higher prevalence of comorbidities, and a lower prevalence of drug addiction, although without significant differences. Alcoholism was significantly associated with LTBI (16.1% vs. 3.8%, *p* = 0.001). Among the 31 PLHIV diagnosed with LTBI, one was lost to follow-up after diagnosis, and one deceased during the hospital stay that led to LTBI diagnosis, while chemoprophylaxis was recommended for 28 of the remaining individuals. Of these, 26 agreed to start chemoprophylaxis immediately, while two opted to commence treatment for LTBI in the future. The process of LTBI screening is visualized in Figure 3 via a Sankey plot.

Among the remaining PLHIV with LTBI (n = 29), 16 were on tenofovir alafenamide (TAF)-based regimens, 1 was on a tenofovir disoproxil fumarate (TDF)-based regimen, and 12 were on tenofovir-free regimens. Specifically, 22 were on integrase strand transfer inhibitor (INSTI)-based regimens, 3 were on protease inhibitor (PI)-based regimens, 2 were on PI and INSTI-based regimens, 1 was on a combined regimen with non-nucleoside reverse transcriptase inhibitor (NNRTI) and INSTI, and 1 was on an NNRTI-based regimen. The majority of the LTBI therapy consisted of 6–9 months of isoniazid (25/26, 96.15%), while 1 patient received rifampin plus isoniazid. In only one case was ART changed before starting LTBI chemoprophylaxis. Transaminases and bilirubin levels were monitored in all PLHIV before and during treatment (in Table 2, where treatment was still ongoing, we used the last observed value).

A total of 18 completed the therapy, while 3 discontinued it and 5 were still on treatment at the time of the analysis. One patient refused to continue after starting treatment, while the other two experienced adverse events (both were Italy-born). Both adverse events occurred in patients taking isoniazid, with one developing acute liver disease and the other experiencing a transient ischemic attack shortly after initiating therapy and subsequently declining to resume chemoprophylaxis. Moderate and transitory increases in transaminases (<3 normal values) were registered in 4 cases, all with normal values at the end of the therapy. 

## 4. Discussion

Our multicenter study reported retrospective and prospective data about the screening and prevalence of LTBI among PLHIV in Italy. Another 1000 PLHIV underwent QFT TB Gold Plus testing across five Italian centers, revealing a 3.4% prevalence of positive results among PLHIV without a history of previous TB. QFT-TB Gold Plus demonstrated reliability for TB screening in PLHIV. Elisa Petruccioli et al. demonstrated that the CD4 count did not influence the distribution of IFN-γ values in HIV–TB and HIV–LTBI patients. Furthermore, cytometry results demonstrated that HIV infection reduced the CD4+ T-cell response but did not impact the CD8+ T-cell response, which likely compensates for the CD4-response impairment related to HIV infection [11]. Symptoms, radiography, CT, and smear microscopy were employed to exclude subclinical TB, resulting in the diagnosis of one case. However, clinical discrimination between LTBI and active TB remains challenging, particularly in PLHIV, where clinical manifestations can be masked, and active tuberculosis can occur even in the absence of lung lesions. Previous studies have explored antibodies and new *Mycobacterium tuberculosis*-specific immunologic antigens to differentiate LTBI and active TB [12]. Additionally, plasma CCL1 and IL-2Ra have been considered as potential biomarkers for distinguishing active TB from LTBI in low-TB-burden settings unaffected by HIV infection [13]. Excluding the single case of subclinical tuberculosis diagnosed in our data, among the 37 PLHIV with positive results, 7 were lost to follow-up; ultimately, only 28 were offered chemoprophylaxis. As observed in a previous study, there were substantial losses in the LTBI cascade-of-care before treatment initiation and among cohorts utilizing LTBI tests; thus, the cumulative proportions of PLHIV starting and completing TB preventive therapy were 60.4% and 41.9%, respectively [14]. The overall prevalence of LTBI in our study was 2.8%, although it could be slightly higher (3.3%) (37/1105) if we considered that six individuals with positive results were lost before differentiating the type of TB. This prevalence is lower compared to others reported in the literature for PLHIV. Meaghan M Kall et al. reported an LTBI prevalence of 9.2% (46/502, United Kingdom), Anne Bourgarit et al. reported an LTBI prevalence of 11.5% (47/407, France), Helena A. White et al. reported an LTBI prevalence of 11.1% (117/1053, United Kingdom), and Tessa Runels et al. reported an LTBI prevalence of 5.8% (11/189, United States of America) [15,16,17,18]. An Italian survey indicated a prevalence of 6.5% (32/495) [8]; however, LTBI screening in PLHIV was not uniformly performed in all investigated centers. Elzbieta Matulyte et al. noted that the prevalence of LTBI among PLHIV was higher compared to the HIV-uninfected population and found an association with intravenous drug use [19]. In our data, no significant difference was observed, with 16.1% (5/31) of PLHIV with LTBI addicted to drugs, while 11% (119/1056) of those with a negative result were addicted. Although our data are limited, we found a positive association with alcoholism. However, we found a positive IGRA test association with a non-Italian country of origin, with most of the non-Italy-born screened individuals coming from Africa. Other studies conducted in low-prevalence countries have shown an association between country of origin and positive results during LTBI screening in PLHIV [15,16,17]. Overall, the global prevalence of LTBI was found to be 24.8%. specifically, Southeast Asia emerged as the region with the highest LTBI prevalence, followed by Africa, with a prevalence of 26.6% [20]. These data align with the epidemiological landscape of TB, as highlighted by WHO, which reported Africa as the region with the second-highest TB incidence after the southeast Asia region [1]. In the latest WHO report, approximately 23% of new estimated TB cases were attributed to the African region [1]. These statistics validate our findings, which suggest a higher prevalence of LTBI among PLHIV originating from Africa, which represented the majority of non-Italy-born PLHIV screened in our study. In our study, more than 90% of PLHIV diagnosed with LTBI were recommended chemoprophylaxis, 92.8% (26/28) initiated it, and 88.4% (23/26) completed it or were still on treatment at the time of this analysis. In almost all cases, isoniazid therapy was administered for a duration of 6–9 months, as determined by the clinician. Only one case of severe hepatic injury related to isoniazid was reported, and another patient experienced a transient ischemic attack, likely unrelated to the therapy. Regarding the initiation of chemoprophylaxis, the prevalence reported in previous studies [8,15,16,17,18] ranged from 28% [16] to 87% [15]. Although WHO guidelines advocate for LTBI treatment in all PLHIV [1], the inadequate implementation of this recommendation may stem from skepticism regarding the effectiveness of treatments, the low incidence of TB in developed countries, concerns about treatment adherence, safety issues, and potential interactions with ART. Our study reported very few adverse events; of these, only one was definitively associated with LTBI treatment. Additionally, ART was changed in only one case. Isoniazid did not interact with commonly used ART regimens, while rifampin interacted with many HIV drugs, such as TAF, dolutegravir, and boosted/PI. In contrast to our data, a previous study highlighted a higher number of adverse events (33.3%), mainly hepatotoxicity, in PLHIV taking isoniazid for LTBI [21]. Further prospective studies are warranted to elucidate this matter. A reasonable compromise is the regular monitoring of hepatitis markers during therapy to promptly suspend therapy, if necessary. PLHIV have a higher prevalence of LTBI, and data in the literature demonstrate that chemoprophylaxis reduces TB incidence in this group [3]. Additionally, Tecla M. Temu et al. found heightened levels of pro- and anti-inflammatory cytokines among PLHIV with LTBI in a study comprising four groups of PLHIV with LTBI and without LTBI and HIV-uninfected individuals with and without LTBI [22]. The treatment of LTBI could reduce these markers.

## 5. Conclusions

In conclusion, our multicenter study provides valuable insights into the prevalence of LTBI among PLHIV in Italy, as well as the outcomes of LTBI screening and therapy initiation. The study revealed a prevalence of LTBI of 2.8% among PLHIV, with non-Italy-born individuals showing a significantly higher likelihood of testing positive for LTBI. Despite the recommendations for LTBI treatment in all PLHIV, our findings indicate that there are substantial gaps in the implementation of chemoprophylaxis, with only 26 PLHIV with positive result initiating therapy and 7 lost to follow-up before of completing the screening or starting chemoprophylaxis. Adherence to LTBI therapy was relatively high, with only 3 individuals discontinuing the prescribed regimen. However, adverse events associated with LTBI treatment, particularly hepatotoxicity, underscore the importance of careful monitoring and management during therapy. Our study highlights the need for enhanced efforts to ensure universal screening for LTBI among PLHIV. Further research is warranted to explore strategies to improve LTBI cascade-of-care outcomes and optimize the safety and effectiveness of LTBI treatment regimens in PLHIV. Future studies should consider multicenter networks involving several Italian centers to increase the number of patients enrolled and to improve the generalizability of our findings. Furthermore, to reduce loss to follow-up in LTBI screening and enhance treatment initiation among PLHIV, it is crucial to provide comprehensive education on the significance of LTBI screening and treatment, as well as on the potential consequences of non-adherence. Shorter treatments could promote better adherence and acceptance of latent TB therapy. However, challenges arise in PLHIV due to pharmacological interactions between rifampin or rifapentine and commonly used integrase-inhibitor-based regimens. Exploring novel anti-tuberculosis drugs for chemoprophylaxis may be essential to address this issue effectively. In addition, studies are needed to explore the reasons for failure to initiate and complete LTBI therapy.

## Figures and Tables

**Table 1:** The table shows the features of PLHIV with LTBI compared with PLHIV who tested negative by IGRA.

Variables	All (n = 1087)	IGRA Positive (n = 31, 2.8%)	IGRA Negative (n = 1056, 97.2%)	*p*
**Sex, n (%)**				0.61
**Female**	254 (23.4)	9 (29.0)	245 (23.2)	
**Male**	817 (75.2)	22 (71.0)	795 (75.3)	
**Other**	16 (1.5)	0	16 (1.5)	
**Country of origin, n (%)**				0.002
**Italy**	892 (82.1)	19 (61.3)	873 (82.7)	
**Non-Italy-born**	195 (17.9)	12 (38.7)	183 (17.3)	
**Age, median (IQR)**	49 (40–56)	49 (35–57)	48.5 (40–56)	0.90
**Drug users, n (%)**				0.64
**No**	842 (77.5)	22 (71.0)	820 (77.6)	
**Yes**	124 (11.4)	5 (16.1)	119 (11.3)	
**Missing**	121 (11.1)	4 (12.9)	117 (11.1)	
**MSM, n (%)**				0.75
**No**	686 (63.1)	18 (58.1)	668 (63.3)	
**Yes**	270 (24.8)	8 (25.8)	262 (24.8)	
**Missing**	131 (12.1)	5 (16.1)	126 (11.9)	
**Alcoholism, n (%)**				0.001
**No**	619 (57.0)	19 (61.3)	600 (56.8)	
**Yes**	45 (4.1)	5 (16.1)	40 (3.8)	
**Missing**	423 (38.9)	7 (22.6)	416 (39.4)	
**Years from HIV diagnosis, median (IQR)**	7.2 (2.3–17.3)	5.0 (1.1–13.9)	7.3 (2.3–17.3)	0.34
**CD4, median (IQR)**	618 (426–840)	647 (460–802)	617 (426–841)	0.57
**Nadir CD4, median (IQR)**	300 (104–484)	406 (275–570)	300 (103–482)	0.06
**Comorbidities, n (%)**				0.16
**0**	786 (72.3)	26 (83.9)	760 (72.0)	
**1**	256 (23.6)	3 (9.7)	253 (24.0)	
**≥2**	45 (4.1)	2 (6.4)	43 (4.1)	
**Cardiovascular diseases, n (%)**	239 (22.0)	4 (12.9)	235 (22.2)	0.27
**Diabetes, n (%)**	61 (5.6)	1 (3.2)	60 (5.7)	1.00
**Solid neoplasia, n (%)**	40 (3.7)	1 (3.2)	39 (3.7)	1.00
**Hematologic malignancy, n (%)**	10 (0.9)	1 (3.2)	9 (0.8)	0.25

**Table 2:** Follow-up data of PLHIV who started LTBI chemoprophylaxis.

Variables	All (n = 26)	Italy-Born (n = 14)	Non-Italy-Born (n = 12)	*p*
Pretreatment				
Ast Median IQR	18 (14–21)	17 (14–19)	22 (16–25)	0.10
Alt Median IQR	18 (15–22)	17 (15–21)	18 (15–24)	0.54
Bilirubin Median IQR	0.40 (0.29–0.52)	0.39 (0.29–0.50)	0.52 (0.28–0.80)	0.48
Last measured value *				
Ast Median IQR	21 (18–25)	21 (18–29)	21 (17–24)	0.68
Alt Median IQR	22 (13–27)	26 (15–30)	17 (13–23)	0.09
Bilirubin Median IQR	0.41 (0.34–0.72)	0.54 (0.33–0.72)	0.39 (0.35–0.41)	0.48

* The last one was frequently taken before the therapy ending. Ast = aspartate aminotransferase, Alt = alanine aminotransferase.

## Data Availability

The data that support the findings of this study are available from the corresponding author, AC, upon reasonable request.
